# A Review on Passive and Integrated Near-Field Microwave Biosensors

**DOI:** 10.3390/bios7040042

**Published:** 2017-09-23

**Authors:** Subhajit Guha, Farabi Ibne Jamal, Christian Wenger

**Affiliations:** IHP, Im Technologiepark 25, 15236 Frankfurt (Oder), Germany; jamal@ihp-microelectronics.com (F.I.J.); wenger@ihp-microelectronics.com (C.W.)

**Keywords:** biosensor, microwave biosensor, high frequency biosensor, dielectric sensor, dielectric spectroscopy, semiconductor, CMOS biosensor

## Abstract

In this paper we review the advancement of passive and integrated microwave biosensors. The interaction of microwave with biological material is discussed in this paper. Passive microwave biosensors are microwave structures, which are fabricated on a substrate and are used for sensing biological materials. On the other hand, integrated biosensors are microwave structures fabricated in standard semiconductor technology platform (CMOS or BiCMOS). The CMOS or BiCMOS sensor technology offers a more compact sensing approach which has the potential in the future for point of care testing systems. Various applications of the passive and the integrated sensors have been discussed in this review paper.

## 1. Introduction

**Figure 1:**
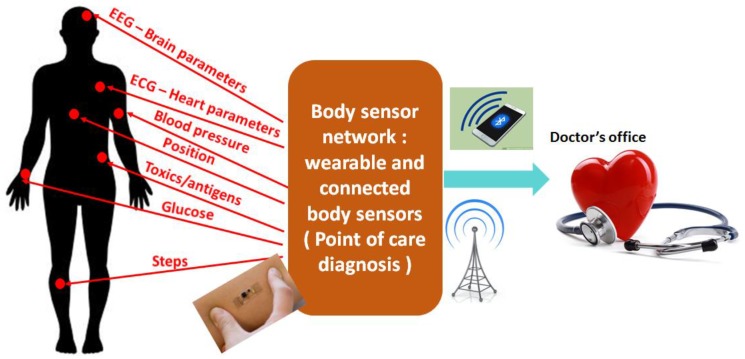
Point of care testing (POCT) system. Inspection of vital body parameters can be performed at the patient site and the data can be transmitted using connected devices.

By the end of 2025, a large section of the developed or developing countries will encounter a population whose average age is above sixty-five [1,2]. The dramatic impact of the ageing population is directly translated to the health care sector of the society, calling for an urgent need for disruptive technology. There has been substantial investigation and research in the area of point of care testing (POCT) technique, in order to establish rapid and fast detection platforms for healthcare. POCT technique involves tests that are conducted in non-laboratory conditions, primarily involving self-testing by the patient. With the strong impact of the ageing society in Europe [3], America [4], Asia [5], the establishment of rapid and cheap POCT technology, along with high accuracy of detection of biological parameters, will serve as a boon, not only to the consumers or patients, but as well as to the doctors. Additionally, the advent of connected devices and internet of things (IOT) will make the POCT devices ubiquitous. Therefore, POCT together with the aid of connected devices have the potential to usher in the disruptive technology that the healthcare sector is aiming for. One of the primary aims of establishing POCT devices is to bring down the overall time required to produce the diagnostic test results [6,7,8], along with easy handling of medical test devices. At the same time, keeping the cost of POCT systems low is also of prime significance [7], in order to address a mass-market. Figure 1 shows the use of POCT in a “connected devices” environment. A body sensor network comprising of “off body” sensors and wearable medical devices will lead to continuous monitoring of vital body parameters, like blood pressure, glucose content in blood, toxic contents, brain activities, and more.

**Figure 2:**
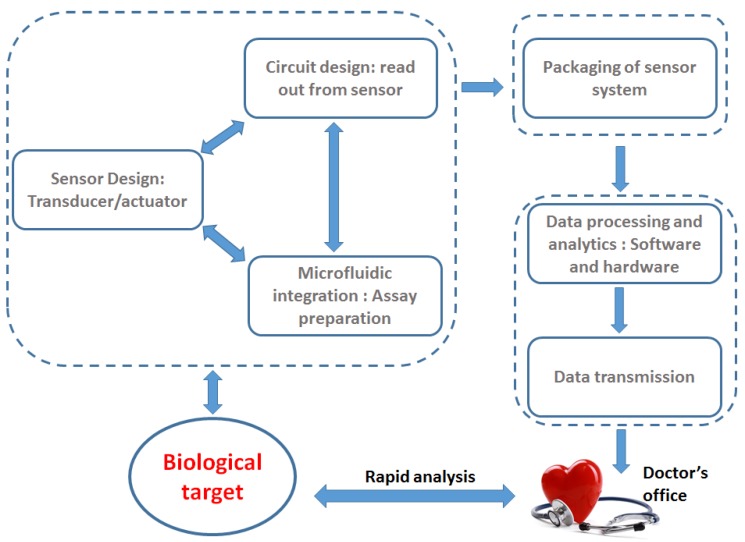
Key features of POCT system. The efficient design of POCT system requires the interaction of life science, chemistry, microsystems, sensor design, circuit design, packaging, and data analytics.

In a connected device environment, the data obtained from continuous monitoring can be simultaneously fed to the doctor’s office, thus, having the capability of raising the alarm for any abnormality in health parameters. This enables rapid diagnosis of medical conditions and fast treatment of the same, which is the goal for high healthcare standard for the societies in developed as well as developing nations. Developing sensitive and fast biomedical analysis systems is the foundation for such POCT systems. The design of cheap POCT systems with an extremely sensitive bio-analysis unit calls for the convergence of various research domains ranging from life science, chemistry, sensor design, circuit design, microfabrication, system design, and more. The various aspects of the POCT are shown in Figure 2.

The sensor system is designed to detect the target biological parameter. As shown in Figure 2, the sensor system includes a sensor or a transducer for the detection of the biological parameter. However, along with the sensor, the overall system requires circuit design for the sensor operation and reading out of the sensor data. Additionally, for the design of the sensor system, microfluidic integration and preparation of chemical assays are required for handling of biological samples. In order to design integrated sensor systems for POCT, the handling of biological samples plays a key role in determining the circuit design approach. This can be attributed to the fact that the biological target should interact only with the sensor, and not the additional circuits. Therefore, there is a close relationship between the “circuit and sensor design” and the “microfluidic and chemical assay design”. The packaging requires a close understanding of the overall sensor system, such that the sensitivity is not lost and additionally does not add noise to the involved circuits. The final block is designed for the transmission of the sensor data. Design and implementation of high precision sensor is a crucial step for the establishment of POCT system. Considerable amount of research work has been dedicated to establishing such high sensitive sensors, also called biosensors or chem–bio sensors. A biosensor is a device that is used to detect and quantify biological targets based on a biochemical reaction [8] or based on intrinsic properties of the biological target material. The biological target can be biomolecules, like proteins, DNA, biomarkers, pathogenic organisms, hormones, or other medically relevant analytes like glucose, medical parameters like pulse, heart-beat, etc. Optical biosensors have been worked on for a long time [9,10,11,12,13,14,15], and some of the sensor schemes have already been realized as a commercial product. One such example is the pulse oximeter based on optical absorption principles [16] measuring proportion of oxygenated hemoglobin in blood. Commercially available pulse oximeters are hand held devices showing a classical example of POCT system. The success of such clinical diagnostic devices led to the research of optical biosensors in the area of biomarker detection, like immunosensors [17], cytometric applications [18], proteomic analysis [19], infectious disease diagnostics [20], etc. Most of the optical biosensors are based on labeled detection technique [15]. The labeled detection approach is essential for extremely high specificity of the biological targets. Additionally, label free optical techniques like surface enhanced Raman spectroscopy are also popular for biological sensing, like dopamine levels [21]. On the other hand, electrochemical sensors have also been another cornerstone technique for POC diagnostic systems. The evolution of the electrochemical glucose sensor into a viable cheap commercial product, ever since its inception in 1960s, is an example of the success of electrochemical techniques. Therefore, electrochemical techniques for biosensors applied now to biomolecule detection or cytometric applications are also being researched for application in POCT systems [22,23,24]. The technique of sensing applied for biological purposes is application specific and is therefore, difficult to establish a universal biosensing technique for POCT device. While optical sensing technique is feasible for a vast number of applications like pulse oximeter and ELISA (enzyme linked immunosorbent assay) technique, electrochemical sensing is suited for other applications, like blood analysis monitoring systems, like the commercial i-STAT from Abbott Point of Care, USA [25]. Another potential sensing scheme is the use of electromagnetic waves, however, not in the optical frequency range. Electromagnetic waves in the frequency range of GHz to THz are extremely sensitive to the changes in the water content in a sample, and can be successfully used for detection of biological targets based on changes in the water content. 

Recently, biosensors operating in the microwave frequency range (1 GHz–100 GHz) of the electromagnetic spectrum have gained considerable interest. The interaction of electromagnetic wave in this frequency range with biological material can be utilized to design highly sensitive sensors. The advances in microwave engineering have led research groups to explore high frequency characterization of biological suspensions and biomaterials. In this review paper, recent advances in near-field microwave biosensors have been addressed. The paper will address, specifically, near-field microwave biosensors. As the name indicates, near-field microwave biosensors have the probe biomaterial in close proximity of the sensor (the distance of the biological target to the sensor is less than the wavelength of the sensor operation). The near-field biosensing is an interesting and efficient approach for in vivo biosensors. The sensor being in close proximity to the probe sample ensures high sensitivity. On the other hand, a far-field sensing approach is beneficial for characterization of samples showing absorption of electromagnetic waves at particular frequencies in the GHz to THz electromagnetic spectrum. Additionally, near-field microwave biosensors offer the advantage of usage of extremely small sample volumes, due to the small sensor size. The near-field microwave biosensors can be distinguished into passive microwave structures, which are not integrated on semiconductor technology platform—complementary metal oxide semiconductor (CMOS) or bipolar complementary metal oxide semiconductor (BiCMOS) technology. The other type includes integrated sensor platform, where the sensor is integrated with circuits on CMOS or BiCMOS technology. This review paper addresses both passive microwave biosensors and integrated microwave biosensors for various applications. 

## 2. Microwave Biosensors 

### Microwave Interaction with Biomaterial

Water is the principle constituent of biological materials, for example, the water content in human erythrocytes vary from a minimum of 55% to a maximum of 75%, as reported by Kageyama et al. [26]. The same is the case for biological suspensions, where biomolecules are suspended in aqueous medium; therefore, water governs the maximum fraction of the suspension. The same is observed for human tissues, and organs [27], where water is the dominating constituent. Therefore, interaction of electromagnetic waves in the microwave frequency range with water is the principle primarily utilized for the establishment of the microwave biosensor [28]. 

The interaction of electromagnetic waves with a medium is characterized by a phenomenon called polarization. The propagation of the electromagnetic wave causes reorientation of the molecules of the medium, causing a momentary segregation of positive and negative charges in the medium. This phenomena is called polarization. The phenomenon of polarization is characterized by a medium parameter termed as the “permittivity”, and is a frequency dependent property [29]. The reorientation of the molecules is observed up to a certain frequency, and beyond this frequency, the molecules are unable to follow the electromagnetic field for reorienting. This gives rise to the frequency dependency of permittivity. Water being a polar liquid, the same phenomenon of polarization is observed for an interaction with an electromagnetic wave. The permittivity of the water can be modeled using Debye relaxation mechanism [30,31]. The dielectric permittivity of water due to the single Debye mechanism reduces with frequency. The dispersion mechanism is given by the equation,

(1) $$\varepsilon_{f}=\varepsilon_{\infty }+\frac{\varepsilon_{s}-\varepsilon_{\infty }}{1+{\left(\frac{f}{f_{c}}\right)}^{2}}$$

**Figure 3:**
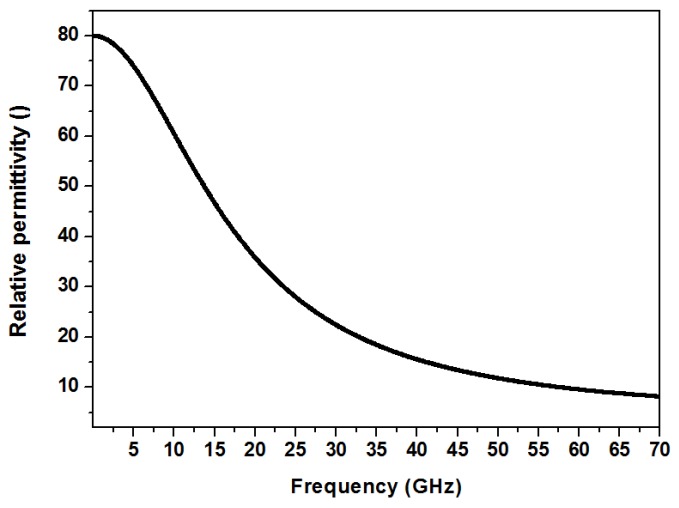
Dielectric permittivity of water as a function of frequency. The characteristic frequency of the single Debye relaxation is 17 GHz.

*ε_f_* is the permittivity at the operating frequency *f*. *ε_s_* and *ε*_∞_ are the static and infinite frequency permittivity, respectively. The characteristic frequency of the relaxation mechanism is given by *f_c_*. The variation of the permittivity of water with respect to frequency is well studied [30]. The variation of the permittivity of water with respect to frequency is shown in Figure 3. 

At low frequency, where the oscillations of the dipoles can follow the applied frequency of the electric field, the permittivity of water at room temperature is 80. The relaxation frequency of a water molecule is 17 GHz. The dielectric permittivity decreases around this frequency. At extremely high frequency (around 200 GHz), the permittivity is approximately 4. 

The operating frequency of microwave biosensors can range from 1 GHz to 100 GHz. Most of the microwave biosensors operate in the frequency range where the permittivity of water is considerably high as compared to the biomaterial. This makes the biosensor operate in the frequency primarily in the range of 1 GHz to 30 GHz. The choice of biosensors operating in this range also stems from the limitations of the biosensors operating in the megahertz region. Electrochemical sensors based on impedance measurements at the frequency range of MHz have been demonstrated by various research groups, like Goh and Ram [32], Krommenhoek et al. [33], and Faenza et al. [34]. Commercial products based on electrochemical impedance spectroscopy have been demonstrated by Micronit microtechnologies [35], Gamry Instruments [36], and more. In this frequency range (“low-frequency”), biological suspensions, especially of suspended cells, show dielectric dispersions based on their properties, for example, potential across the cell membrane and cell walls [34], or double layer capacitance between the electrode and the suspension, and more. Therefore, the impedance based sensors operating in the megahertz range are useful for characterizing properties of cell wall and cell membranes in biological samples. However, in detection of concentration of biomarkers, pathogens, concentration or type of cells, microwave (GHz range) dielectric sensing is an extremely attractive option. This is caused by the permittivity of water, which has a contrasting difference compared to the permittivity of biomaterials. For example, in biological suspension, the change in the concentration of biomolecules, in a given volume of the suspension, changes the influence of the suspending medium, which is primarily water, which influences the permittivity substantially. A similar example can be obtained in detection of living or dead cells. Dead cells have a different concentration of water as compared to living cells, thus providing permittivity contrast, which is required for sensing. 

Research in the microwave biosensor community can be classified as “passive sensors” and “integrated sensors”. Microwave components, like transmission lines (microstrip and coplanar), lumped capacitors, waveguides, fabricated on a substrate and used for detection of biological materials, can be termed as “passive sensors”. On the other hand, there are research groups working on integrating microwave sensor structures on standard semiconductor (CMOS/BiCMOS) technology platform. Such sensors can be termed as “integrated biosensors”. The purpose of this review is to address specific biological applications that have been recently addressed using these two sensor approaches.

## 3. Passive Biosensors

As early as 1998, Stuchly et al. [37] demonstrated biosensors based on waveguide structures. Since then, considerable amounts of research work have been devoted to the establishment of microwave biosensors. Sensors based on whispering gallery mode resonator [38], coaxial resonator [39], coplanar lines [40,41], and capacitors [42], have been demonstrated for the detection of cells and proteins, DNAs, biomarkers for tumors, and cancer, etc. Interferometric microwave sensors for detection of biological cells have also been shown in [43].

**Figure 4:**
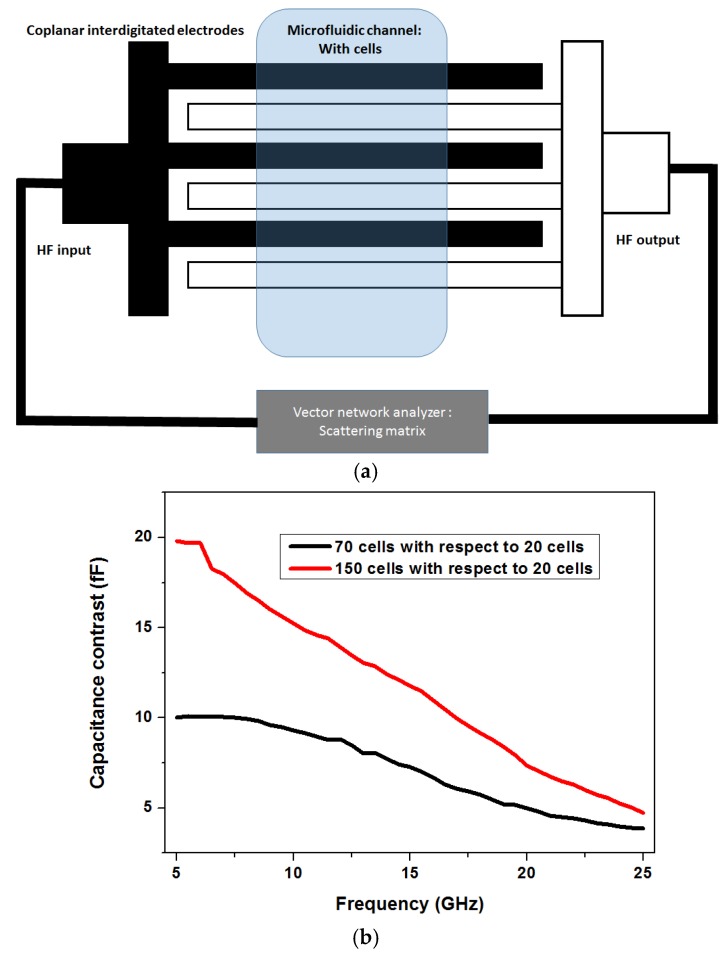
(**a**) Coplanar passive interdigitated capacitor on a microwave substrate integrated with microfluidics; (**b**) Distinction of concentration of cells using the capacitor sensor [42].

The detection of concentration of particles, for e.g. cells in a medium, is extremely significant as it can aid in screening of unwanted bio material in excess amount. Figure 4 shows one such approach to detect concentration of cells in a solution used by Grenier et al. [42,44]. A coplanar multi-fingered capacitor is used as a passive sensor structure, in order to detect different concentrations of cells. 

The electric fields between the fingers of the capacitors that penetrate into the material under test are used for the sensing purpose. The capacitive sensor is fabricated using metal electrodes on a microwave substrate and a polymer microfluidic system is integrated on top of the sensor. The advantages of microwave sensing towards miniaturization can be understood from the sensor setup as shown in Figure 4a. The dimension of the sensor which is in the order of micrometers matches the dimensions of the cells being investigated. The measurement setup in such a sensor system involves measuring of the scattering parameters (also called the s-parameters) [29], of the passive capacitor. This uses a typical network analyzer measuring s-parameters in two ports or a single port configuration. The s-parameter matrix can be used to obtain the impedance of the microwave structure (capacitor here). 

Figure 4b shows the approximate capacitance contrast obtained, when the sensor was loaded with biological suspensions with cells with respect to the normalized measurement when the sensor had 20 yeast cells on top of it. As seen in the measurement, the capacitance contrast is higher at the frequency range of 5 GHz to 10 GHz. The contrast reduces at high frequencies. As mentioned in the previous section, this is attributed to the strong permittivity difference between the cells and the aqueous medium at the frequency range of 5 GHz to 15 GHz. 

**Figure 5:**
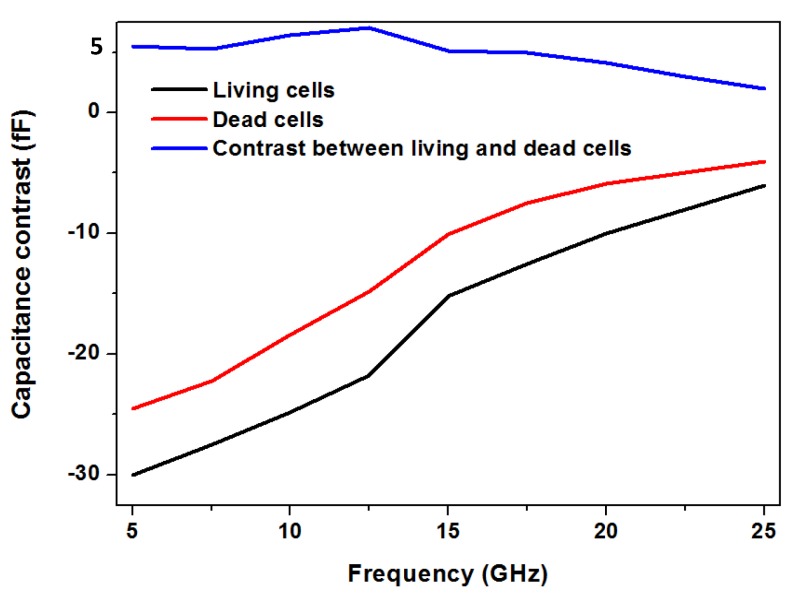
Distinction of living and dead cells using coplanar capacitive sensors [44].

The same group has also shown differentiation of living and dead RL lymphoma cells using the broadband spectroscopy measurement [44] employing the capacitive sensor. This is approximately illustrated in Figure 5. A crucial feature addressed in this work is the “label-free” nature of microwave sensing. The distinction between living and dead cells can be achieved by staining the cells followed by fluorescence spectroscopy. However, in case of microwave sensing, no additional labeling mechanism is required.

Another often used configuration for microwave detection technique is based on interferometry. Sensitivity of the order of single cells has been demonstrated by Yang et al. [45]. 

**Figure 6:**
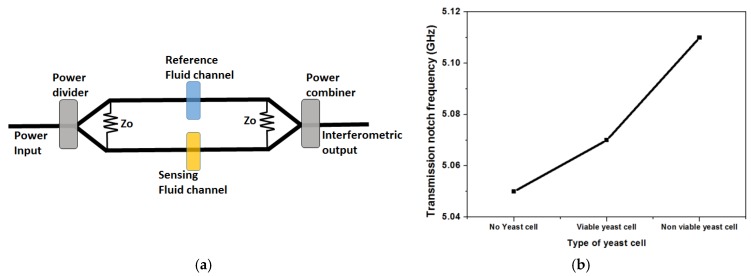
(**a**) Interferometric design of passive transmission lines for biosensing; (**b**) Distinction between viable and non-viable yeast cells using the interferometric approach.

The configuration of the sensor is shown in Figure 6a. The interferometric approach, operating at 5 GHz, offers very high sensitivity caused by a differential measurement technique. The sensor architecture, which involves coplanar transmission lines, is fabricated on a microwave substrate. A similar scattering parameter measurement is employed, as discussed, in case of the capacitive sensor. The measurement principle is the same as described above, using s-parameters. The measured s-parameter between port 1 and port 2 (S21: transmission), shows a resonant feature at 5.05 GHz without yeast cells, where there is a dip in the s-parameter transmission curve. The resonant frequency shifts with viable and non-viable yeast cells on the sensor. A similar interferometric sensor concept was used in [43], however, at 1.6 GHz for detection of biological cells. The interferometric sensor, has a differential sensing scheme, with two arms of a microwave power divider. One arm is used as the reference arm while the other arm is used as the sensing arm, also shown in Figure 6a. An approximate distinction between viable and non-viable yeast cells is shown in Figure 6b [45]. This differential measurement gives a high sensitivity, enabling the capability of detection of single cells. 

Whispering gallery mode (WGM) resonators are another popular microwave structure employed for biosensing purposes [46,47]. The WGM resonator is an extremely sensitive technique, due to sharp linewidth of the resonant feature. The theory of WGM resonator can be understood from the theory of cavity resonators [29]. The resonance frequency of the resonator is tuned, due to the presence of biological material on top of it. High sensitivity and detection of extremely small volumes of biological samples can be measured using this sensing approach.

Passive microwave biosensors can be designed and fabricated extremely fast, as it does not require any typical semiconductor fabrication technology. Therefore, for rapid proof of principle of microwave biosensing for a specific application, passive microwave biosensors are an excellent choice. On the other hand, the measurement setup for passive sensors require bulky network analyzers; thus, the target for real miniaturization of sensor systems for application in POCT technology is difficult to achieve. Therefore, integrating microwave biosensors on a standard semiconductor platform is called for. This will enable on-chip signal processing capabilities, leading to simpler output signals like digital voltage values.

## 4. Integrated Microwave Biosensor

The growth of semiconductor industry can be tracked back to the famous paper published by the INTEL co-founder Gordon Moore in 1965 [48]. It was stated in the paper “the number of components that could be incorporated per integrated circuit would increase exponentially with time” [49]. Known as the Moore’s law, this is purely an empirical law, and has successfully continued for more than 50 years of evolution of integrated circuit technology (since 1970), where the number of transistors in an integrated circuit is doubled every eighteen months. As a consequence of this trend, miniaturization of circuits, by scaling down of transistors, has been the driving force for technology advancements. At the same time, there has been considerable advancement in microfabrication technology, leading to the possible fabrication of structures of the order of a few nanometers.

**Figure 7:**
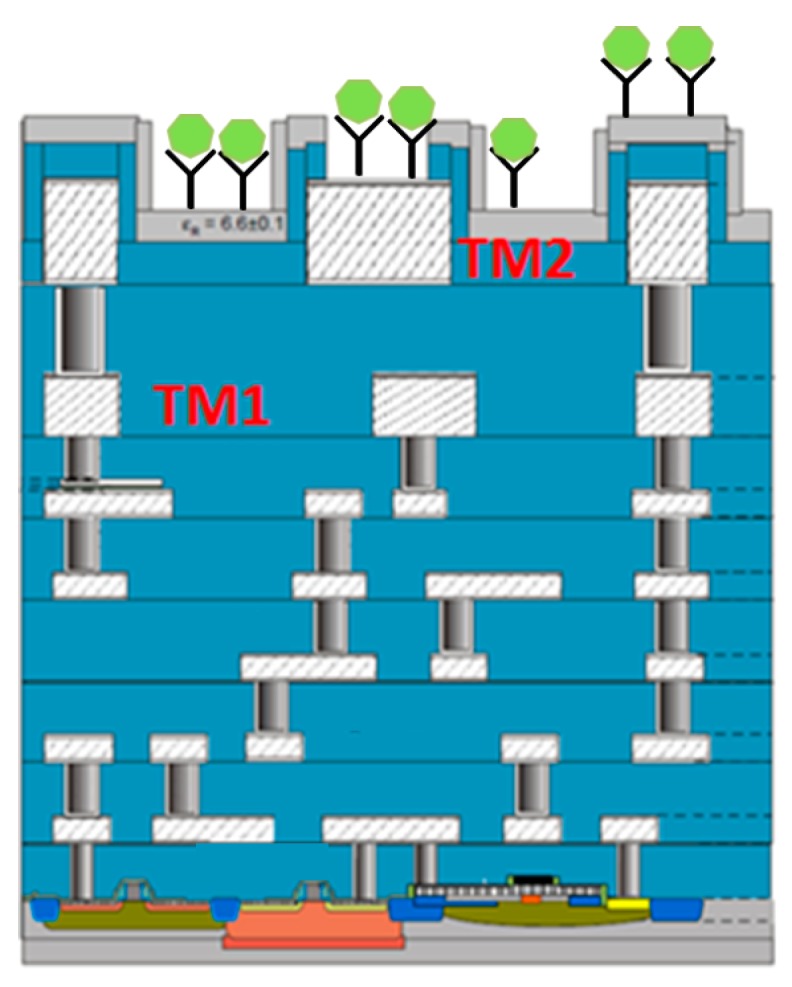
A typical bipolar complementary metal oxide semiconductor (BiCMOS)/complementary metal oxide semiconductor (CMOS) back-end-of-line stack. The top metal layer is used for biomolecule detection.

With this miniaturization of technology node, there is additionally another trend, which is characterized by diversification of functionalities on semiconductor based devices. This trend is referred to as “More Than Moore”. Thus, “More Than Moore” is a heterogeneous integration technology of digital and non-digital applications on the same semiconductor process platform, leading to a wide variety of application fields. Establishment of integrated biochips or biosensors is one such field. The early approach of biosensors used functionalized bio-receptor coupled to a transducer. Further microelectronic circuits were then integrated with the transducer to extract the signal output. This is referred to as the hybrid integrated biosensor; the new approach of complete CMOS biosensor chip involves the elimination of bioreceptors, and capability of using the metal layers of the CMOS process for immobilization or detection of biomolecules. The concept of CMOS based biochips is shown in Figure 7. 

The biomolecules are shown to be captured on the top metal layer (marked as TM2) of the back-end-of-line (BEOL) stack of the CMOS process. Complete CMOS biosensors or biochips have then been explored by researchers for various applications, like DNA characterization [50,51,52], detection of biomarkers [53], and cytometric application [54,55,56]. The next step is the monolithic integration of microwave sensor architecture on the CMOS/BiCMOS platform. Efforts are being made by few contemporary research groups around the world to establish CMOS microwave sensors, but the majority are focused on cytometric applications, like counting of cells [57], or particles based on magnetic makers [58], immunosensing [59], broadband spectroscopy [60] of biological suspensions, and more.

**Figure 8:**
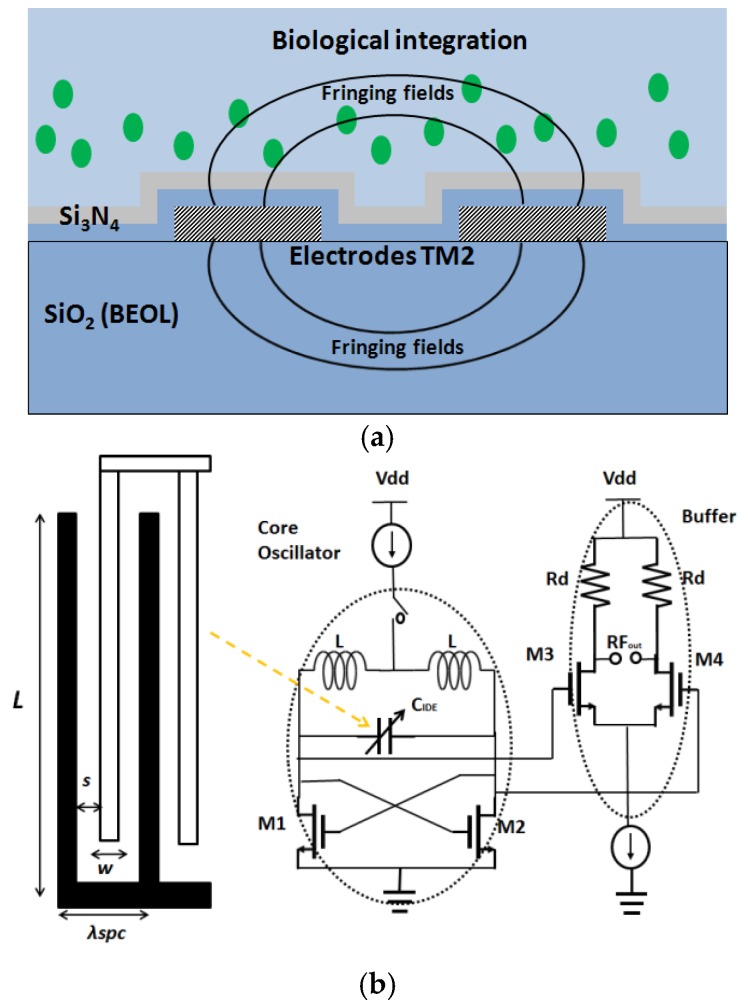
(**a**) Capacitive sensing scheme. The electrodes of the capacitor are fabricated on top metal of the BiCMOS stack; (**b**) The interdigitated capacitor is a part of the cross coupled oscillator.

Our research group has worked on CMOS capacitive sensors operating in the frequency range of 5 GHz to 30 GHz for biological purposes [59,61,62,63,64,65,66,67]. The sensing principle is based on the variation of capacitance embedded in a CMOS oscillator, causing a shift in the resonant frequency of the oscillator. The working principle of a CMOS oscillator can be found in integrated circuit text books [68]. A voltage controlled capacitor (varactor) is used to change the oscillating frequency of the oscillator. In this biosensor approach, the change of capacitance is caused by the variation of fringing electric fields of the sensor structure (interdigitated capacitor or transmission line), due to the change of permittivity on top of it. The sensor capacitor is fabricated on one of the two top metal layers in a BiCMOS process. The schematic representation of the sensing approach, with interdigitated capacitor as the sensor, is shown in Figure 8. 

**Figure 9:**
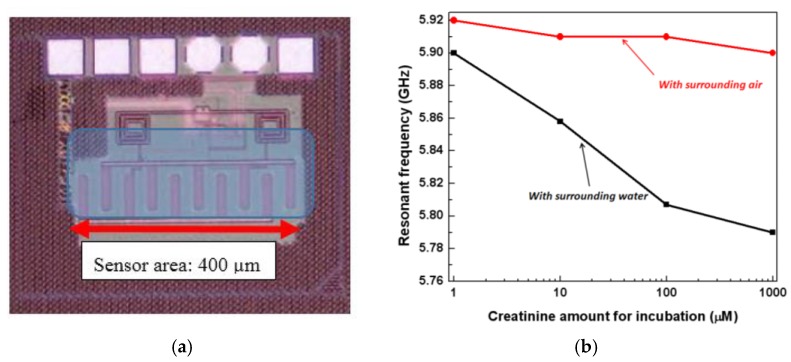
(**a**) The immunosensor chip with the capacitor exposed for biomolecule immobilization; (**b**) Frequency shift of the oscillator for different concentration of creatinine [51].

One of the main applications of the established CMOS biosensor is immunosensing [59]. The immunosensing principle functionality has been demonstrated with a model antibody, creatinine. This work shows the capability of detection of antibodies in the nanomolar concentration range. This is shown in Figure 9.

Figure 9a illustrates the sensor chip. The interdigitated capacitor fabricated on the top metal layer of the BiCMOS stack, is exposed for the immobilization of the antibodies on top of it. The corresponding circuit for the oscillator in which the IDC (Interdigitated capacitor) is embedded is shown. The overall chip size is 0.25 mm^2^. The oscillator output is measured using a spectrum analyzer, and the output pads are shown on the chip. The sensor shows a shift of 35 MHz in the resonant frequency for every 10-fold increase in the concentration of creatinine. 

**Figure 10:**
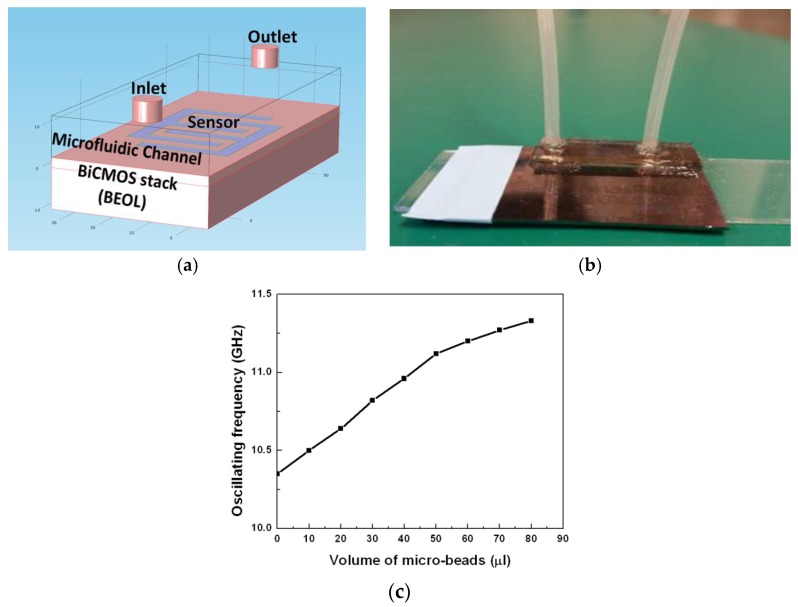
(**a**) Schematic of the microfluidic integration with the CMOS sensor; (**b**) CMOS sensor chip together with the polymer microfluidic; (**c**) Variation of the resonant frequency of the oscillator with different concentrations of particles in a suspension [54].

The same sensing approach has been used for the detection of concentration of particles in the biological suspension [61]. This requires a microfluidic integration with the CMOS sensor chip. Figure 10 shows the CMOS integrated biosensor with the microfluidic system on top. The experiments were conducted using microbeads. 

Figure 10a illustrates the schematic of the microfluidic integration with the CMOS chip, and Figure 10b shows the CMOS sensor chip with the polymer microfluidics on top. It was demonstrated in this work that microbeads of different concentrations suspended in a solution can be distinguished using this sensor. A frequency up-shift of 125 MHz/10 µL increase in bead content in acetone is measured. 

**Figure 11:**
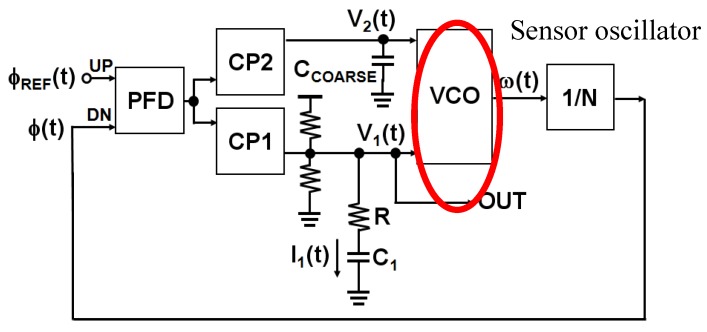
Integration of the frequency shift sensor in a phased lock loop [56].

A similar resonant frequency shift sensor on CMOS platform was used for biosensing approaches by other groups. One such example is shown by Wang et al. [58]. The principle used in this work was the same as described above. The sensor is embedded in an oscillator, however, the sensor used in this work is an inductor, instead of a capacitor. The sensor was shown to operate with magnetic beads. The change of the inductance of the sensor inductor was translated to the oscillating frequency shift of the oscillator. Subsequent work by the same group shows the influence of random noise and noise sources in such a CMOS integrated resonant frequency shift oscillator [69]. Such a noise analysis is extremely significant for complete system integration of such a sensor chip. Such a system integration approach was shown by our group, while integrating the CMOS sensor oscillator in a phase locked loop (PLL), in order to obtain a high sensitivity and a simpler output signal, as compared to the microwave output in the stand alone resonant frequency shift sensor [62]. The target of this work is to obtain an extremely sensitive sensor system with a reasonably easier to handle DC readout of the sensor. The schematic of the overall system is shown in Figure 11. 

The sensor oscillator is embedded in a PLL. The loop is a feedback mechanism which is used to restore the center frequency of the sensor oscillator. The sensor capacitor has two parallel capacitors, and the center frequency can be restored by a controlling voltage on the capacitors, termed as V_1_(t) and V_2_(t). Therefore, the output of the overall sensor system is no longer a microwave signal, but a simpler DC output voltage. To understand the working principle of the overall sensor system in detail, the reader should refer to the work in [62]. Similar PLL based approaches were shown in the work of Nehring et al. [70] and Helmy et al. [71]. In these works as well, the resonant frequency shift sensor was embedded in a PLL. However, the difference lies in the use of number of tuning loops in the PLL. As compared to [70,71], the work in [62] incorporates a dual loop PLL with the two control capacitors, termed as the coarse capacitor and the fine capacitor. Hence, the loops are named as coarse tuning loop and fine tuning loop. The dual loop approach can nullify the influence of the process, voltage and temperature variations while the sensor is operated. 

**Figure 12:**
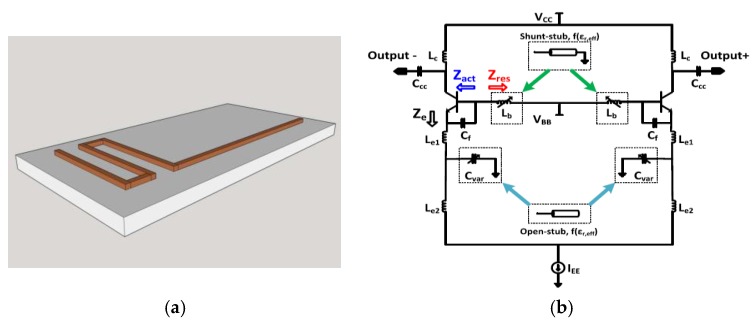
(**a**) Transmission line used as the sensor in the configuration of open or shunt stub; (**b**) Colpitt’s oscillator in which the sensor is embedded [59].

Another CMOS frequency shift oscillator based approach for biosensing was shown in the works of our group [64,65,66]. The sensor is a transmission line configured as an open or a shunt stub [29], and the oscillator topology is different as well, when compared to the above examples. The sensor oscillator is shown in Figure 12. 

**Figure 13:**
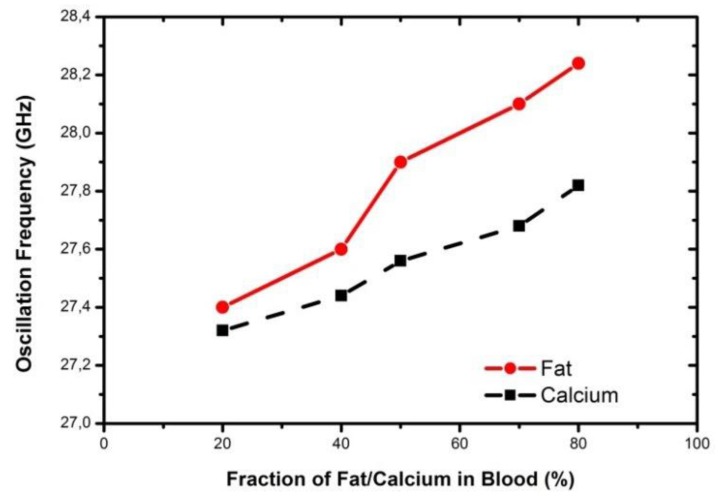
Measurement of fat and calcium in blood using the resonant frequency shift Colpitt’s oscillator with transmission line sensors as stub [66].

The transmission line used as the sensor is shown in Figure 12a. This configuration of the oscillator is called the Colpitt’s oscillator, and is shown in Figure 12b. The transmission line can be used in several configuration (open or shunt stub) in the oscillator topology. The use of the sensor in different topologies was explored in the work [64], and an increase in the sensitivity was shown for different configurations of the sensor in the oscillator. These sensors were also explored to distinguish between two biological targets, like fat and calcium. The results of the sensor approach [66] operating at 30 GHz is shown in Figure 13. Such a sensor is proposed to be used as the first screening sensor for detection of plaque (calcified fat) in human arteries. 

Packaging of the sensor is an important design block for the establishment of a complete POCT system. One such sensor packaging approach was explored by our group in the work [67]. The work shows the packaging and integration of the sensor on a smart catheter platform. Another application of microwave biosensor, explored by our group in the recent times, is the use of the sensor in viscosity sensing [63]. The sensor can be effectively used in determining and characterizing sputum viscosity for early diagnosis of lung disease. Another oscillator approach, based on injection locking oscillator, is explored for flow cytometry [72,73]. The injection locked oscillator is stabilized by a chopper based amplifier; the differential measurement using the injection locking oscillator provides high sensitivity to the sensor approach. All the CMOS integrated frequency shift biosensors described above are single frequency sensors, where a shift in the resonant frequency is observed as an influence of biomaterials on top of the sensor. In all the mentioned cases, the sensors exclusively measure the real part of permittivity. On the other hand, there is considerable research work in order to establish CMOS wideband near-field dielectric spectroscopy systems operating in the microwave frequency range [60,71,74,75,76]. In the work [74], Elkholy et al. describes a digital control oscillator setup for dielectric spectroscopy of biological material at a frequency range of 0.5 GHz to 5 GHz. The work also explains a circuit architecture for determination of the imaginary part of the permittivity. Therefore, it is possible to detect the losses due to the presence of the biomaterials on top of the sensor. The performance of the oscillator based biosensor is dependent on the quality factor of the sensor structure. The quality factor of the sensor structure is degraded by losses in the biomaterial. Water, as mentioned before, forms a large fraction of biomaterial, and the losses in water contribute to the degradation of the quality factor of the sensor [28]. Therefore, the sensor system should be designed in a way such that the degradation due to the losses in the biomaterial does not influence the overall sensor system performance. 

Development of on-chip CMOS vector network analyzer is another approach of performing near-field dielectric spectroscopy for biomaterials at high frequency [77,78]. As mentioned in the case of passive microwave sensors, the s-parameters of the sensors are measured using a network analyzer. There has been an effort to realize such network analyzer on-chip by Nehring et al. [78], Nasr et al. [77]. The use of such an on-chip network analyzer will enable detection of both the real and the imaginary component of the permittivity, and performance of a broadband dielectric spectroscopy of the biomaterial.

## 5. Conclusions

The paper reviewed the establishment and advances made in the near-field microwave biosensing. Microwave biosensing is a substantially new area of research, and is yet to reach the phase of development into commercial products contrary to optical biosensing techniques and electrochemical sensing techniques. However, in certain applications, microwave biosensing definitely offers considerable advantage over optical biosensing or electrochemical biosensing. These primarily involve applications where there is a contrast of water permittivity with respect to the biological material to be sensed. Such applications involve detection of concentration of cells in a suspension, distinction of dead and living cells, immunosensing in an aqueous environment, etc. On the other hand, optical sensors have the advantage of specificity, due to labelling. This makes the optical biosensors extremely sensitive for determination of a specific biomaterial in an aggregation of large number of bio materials. It is still a challenge for microwave biosensors to obtain such specificity. This is due to the fact that a large number of biomaterials can influence the polarization, and in turn, the permittivity of water in a similar way. Therefore, in the case of microwave biosensors, it is essential to know the type of biomaterials that are being detected. This was shown in one of the review examples, where determination of dead and living cells was discussed. 

In this review paper, microwave biosensors were classified into two sections, namely, the passive microwave biosensors, and integrated microwave biosensors. Passive microwave biosensors include stand-alone microwave structures like transmission lines, waveguide resonators, and lumped capacitors, etc., fabricated on a microwave substrate. These passive sensor structures were reviewed in this paper for various applications. Both broadband and single frequency passive microwave biosensors were discussed in this work. The measurement of the passive microwave biosensors involve s-parameter measurements and the use of network analyzers is needed. On the other hand, integrated microwave biosensors are biosensors fabricated in a standard semiconductor technology platform. Integrated microwave biosensors offer the advantage of further miniaturization. With on-chip signal processing capabilities, obtaining a DC output from the microwave sensors was discussed in this paper. This reduces the burden of using cumbersome network analyzers or spectrum analyzers for the measurement. Additionally, in case of batch fabrication, the integrated microwave sensors will be cheap, and thus, aid in mass production. The integrated microwave biosensors were shown to have applications in the area of immunosensing, cytometry, and more. As in the case of passive microwave biosensors, the integrated microwave biosensors were also discussed in terms of single frequency resonator based sensors and broadband sensors. A major challenge for integrated microwave biosensors, especially the ones based on the oscillator topology, is the losses in the biomaterial that degrade the quality factor of the sensor structure. This has been discussed here, and the degradation of the overall sensor performance was pointed out. All in all, “microwave biosensors” is a new area of research and can be a potential alternative to existing biosensing techniques for certain discussed applications in this paper, due to the several advantages described here; however, there are several challenges which need to be surpassed in order to establish microwave biosensing as a standard alternative approach.
